# Nativity in the healthy migrant effect: Evidence from Australia

**DOI:** 10.1016/j.ssmph.2023.101457

**Published:** 2023-06-22

**Authors:** Guogui Huang, Fei Guo, Zhiming Cheng, Lihua Liu, Klaus F. Zimmermann, Lucy Taksa, Massimiliano Tani, Marika Franklin

**Affiliations:** a Centre for Health Systems and Safety Research, Macquarie University, Australia; b Department of Management, Macquarie Business School, Macquarie University, Australia; c Social Policy Research Centre, University of New South Wales, Australia; d Department of Population and Public Health Sciences, Keck School of Medicine, University of Southern California, USA; e Global Labor Organization (GLO), Germany; f UNU-MERIT, Maastricht, The Netherlands; g Deakin University Business School, Deakin University, Australia; h School of Business, University of New South Wales, Australia

**Keywords:** Life expectancy, Healthy life expectancy, Healthy migrant effect, Nativity differences, Cultural similarity

## Abstract

Migrant health constitutes an important public health issue; however, variations in the ‘healthy migrant effect’ among migrants of different nativity are not adequately understood. To fill this gap, this study examines the life expectancy (LE) and healthy life expectancy (HLE) of the Australian-born population and eight major migrant groups in Australia for 2006, 2011 and 2016. The results show that compared with the Australian-born population, the foreign-born population overall had a higher LE and HLE but a lower HLE/LE ratio. Considerable variations in migrant health status according to nativity were also observed. Specifically, migrants from South Africa, Britain and Germany exhibited a similar or higher LE, HLE and HLE/LE ratio, while those from China, India, Italy and Greece had a higher LE but a significantly lower HLE/LE ratio compared with the Australian-born population. Lebanese migrants were the only group who experienced an unchanging LE and a declining HLE from 2006 to 2016. These notable differences in migrants' health outcomes with respect to nativity may be explained by the sociocultural differences between the origin and host countries and the different extents of migration selectivity of different migrant groups. Targeted countermeasures such as improving the quality of life of migrants from culturally diverse backgrounds or with negative migration experiences are suggested.

## Highlights

• Migrants as a whole have a higher LE and HLE than the Australia-born population.
• However, migrants' ratio of HLE/LE stands lower than the Australia-born individuals.
• Considerable nativity variations with respect to migrant's HLE outcomes exist.
• Migrants with West European origin exhibit similar HLE outcomes with Australia-born.
• Asian and South European migrants show a lower HLE/LE ratio than Australian-born.

## 1 Introduction

In recent decades, a growing number of studies have suggested that international migrants tend to be healthier than the native-born populations of their host countries (Anikeeva et al., 2010; Biddle et al., 2007; Cunningham et al., 2008; Vang et al., 2017). Known as the ‘healthy migrant effect’, this phenomenon has been observed in migrant populations as a whole but displays significant variations depending on migrants' countries of origin (Mehta et al., 2016; Reus-Pons et al., 2017; Singh & Miller, 2004). Migrants from specific regions or cultural backgrounds experience better overall health outcomes and a slower deterioration in health compared with migrants from other regions or cultural backgrounds (Anikeeva et al., 2010; Cho et al., 2004; Huijts & Kraaykamp, 2012). Variations in migrant health depend on the socioeconomic and cultural characteristics of both the country of origin and the country of destination, leading to significant health inequalities among migrants (Cunningham et al., 2008; Martinez et al., 2015).

While numerous studies have examined migrant health disparities according to country of birth or nativity (Vang et al., 2017), the existing knowledge on this topic is inadequate. A key limitation in most studies examining the association between nativity and migrant health is the primary focus on mortality, with few paying adequate attention to morbidity or a combination of mortality and morbidity (Mehta et al., 2016; Singh & Miller, 2004). The association between nativity and migrant health outcomes may be different for mortality and morbidity. While it has been shown that migrants have lower rates of mortality regardless of their country of origin (Cho et al., 2004; Cunningham et al., 2008; Vang et al., 2017), the findings on whether migrants from different sociocultural backgrounds have similar morbidity advantages are inconsistent (Anikeeva et al., 2010). This suggests that the current understanding of the association between nativity and migrant health does not extend to morbidity, preventing a full understanding of the health disparities among different migrant groups and warranting further exploration in this field.

Therefore, the present study provides a comprehensive investigation of migrant health in Australia according to nativity with respect to both mortality and morbidity. The life expectancy (LE) and healthy life expectancy (HLE) of eight major Australian migrant groups and the Australian-born population are compared and analysed. Migrant health status is measured using HLE, defined as the average number of years of expected healthy life remaining at a given age if current patterns of mortality and morbidity persist (Jagger & Robine, 2011). HLE incorporates information about both mortality and morbidity (Jagger & Robine, 2011; Stiefel et al., 2010), enabling a comprehensive investigation of the association between nativity and migrant health with respect to both the length and quality of life, which has not been adequately explored in the existing literature.

Despite the growing interest in the association between nativity and migrant health, the research on migrants’ HLE according to nativity has three main limitations. First, prior studies have mostly examined the association between nativity and HLE in migrants as a whole or those from a single ethnic background or country of origin, overlooking disparities in HLE between migrants from different countries of origin (Eschbach et al., 2007; Garcia et al., 2017, 2018, 2019). Moreover, while some studies have categorised countries of origins into different groups, this categorisation tends to be broad (e.g. Western versus non-Western, industrialised versus non-industrialised or English-speaking versus non-English-speaking countries) (McDonald & Kennedy, 2004; Reus-Pons et al., 2017), neglecting the HLE differences between migrants in the same category. Second, previous studies have tended to examine the association between nativity and migrant HLE in a single year, while changes over time have been underinvestigated. An understanding of migrant health according to nativity over time is important to inform policy and address the changing demands for healthcare services. Third, previous research on migrant HLE has focused exclusively on the United States (US), Canada and Western Europe, while, to the best of our knowledge, there is no research on this topic in the Australian context, where migration profiles are different from those of North America and Western Europe. This study responds to these gaps in the literature and provides a comprehensive examination of migrant LE and HLE in Australia according to nativity.

**Fig. 1:**
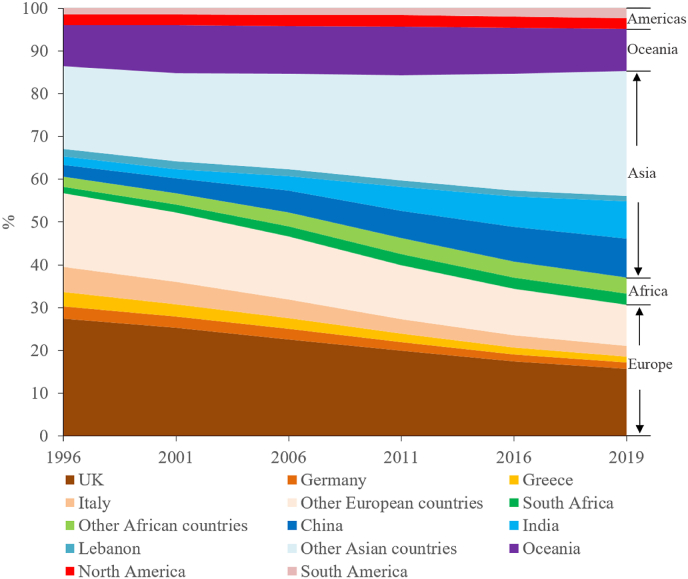
Origins of migrants to Australia: 1996 to 2019.

Source: Australia Bureau of Statistics, hereafter.

This study is set in the Australian context. Australia is a major migrant-receiving nation, and its immigrants have become increasingly ethnically and culturally diverse over the past several decades. While migrants to Australia prior to World War II were almost exclusively from the United Kingdom (UK), in the post–World War II era, migrants from other countries have accounted for a steadily increasing proportion of the Australian population (Richards, 2008). Non-British migration to Australia has occurred in several distinct waves, with Western Europeans (mostly Germans and Italians) arriving in large numbers in the 1950s, followed by Southern Europeans (predominantly Greeks) in the 1960s, Lebanese refugees in the late 1970s and migrants from Indochina, South Asia and East Asia more recently (Krupinski, 1984; Richards, 2008). Consequently, the landscape of migration in Australia has changed profoundly over the past several decades, with a gradually increased proportion of Asian immigrants and a notably decreased proportion of European immigrants, particularly since the 1990s (see Fig. 1). Meanwhile, the proportion of migrants in the Australian population has risen steadily, reaching a peak of 29.7% in 2019 (Australian Bureau of Statistics, 2020).

**Fig. 2:**
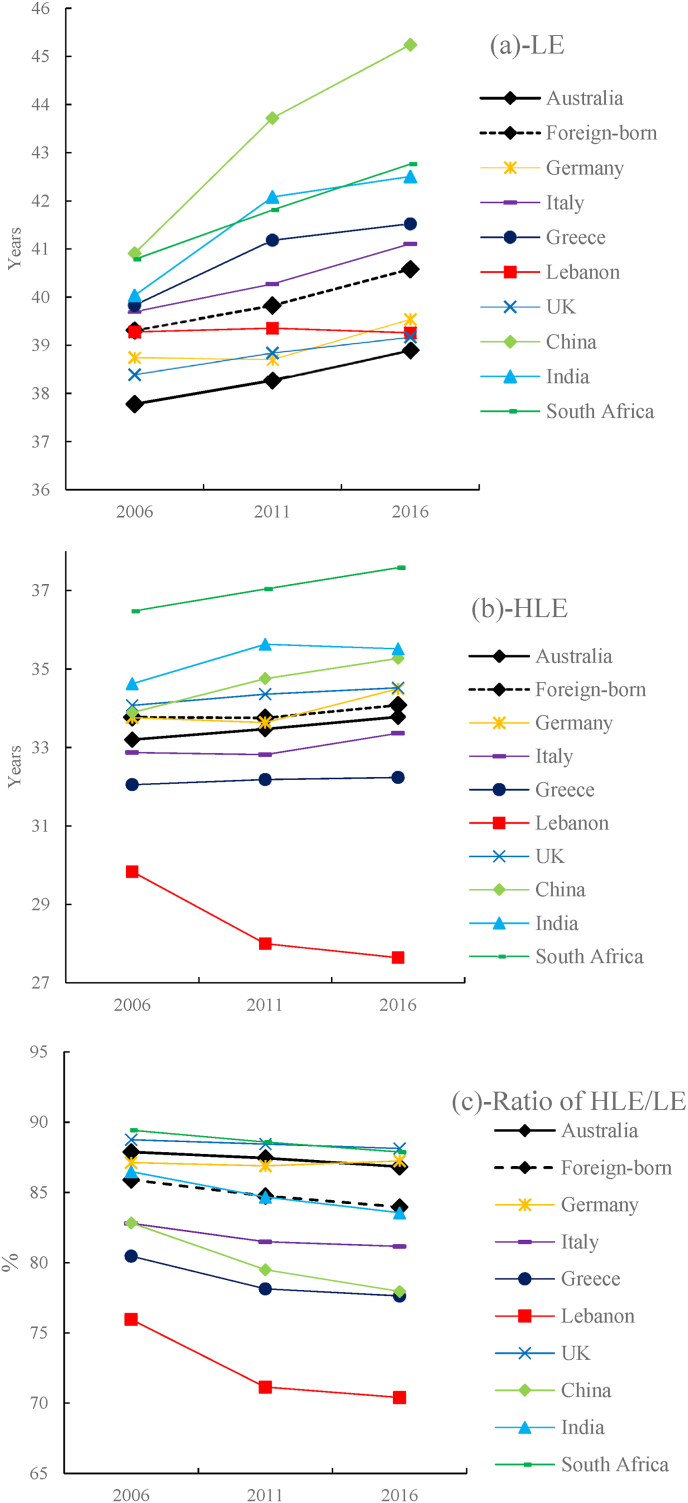
Trends for (a) life expectancy (LE), (b) healthy life expectancy (HLE) and (c) HLE/LE ratio at age 45 for Australians by country of birth: 2006, 2011 and 2016.

In this study, representative migrant groups from each wave of migration to Australia (i.e. those from the UK, Germany, Italy, Greece, Lebanon, India, China and South Africa) are selected. These migrant groups arrived in Australia at various times in history and differ markedly in terms of their original social and physical environments and post-migration socioeconomic and demographic profiles (see Fig. 2). These factors may have had significant effects on their health outcomes (Krupinski, 1984). This study investigates whether and to what extent these migrant groups differ in terms of health and compares this with the health status of the Australian-born population, providing the first exploration of the association between nativity and migrant HLE in the Australian context. The findings shed new light on variations in the healthy migrant effect among different migrant groups and have implications for healthcare policies for migrants in Australia and elsewhere.

## 2 Literature review

### 2.1 The healthy migrant effect and its variations by nativity

A multitude of studies on migrant health and epidemiology have consistently shown that migrants tend to be healthier compared with native populations, particularly with respect to mortality (Anikeeva et al., 2010; Cho et al., 2004; Cunningham et al., 2008; Jatrana et al., 2014; Vang et al., 2017). For example, studies in the US have found that, despite their poor socioeconomic status overall, foreign-born Hispanic Americans have a higher LE compared with US-born, non-Hispanic, Caucasian individuals (Cho et al., 2004). Similar results have been found in Europe, Canada, Australia and elsewhere (Anikeeva et al., 2010; Trovato & Odynak, 2011; Uitenbroek & Verhoeff, 2002). The superior health outcomes of migrants are mainly explained by migration selectivity (i.e. the higher likelihood of healthy individuals migrating successfully), the buffering effect of the original culture (e.g. healthier dietary practices) and the return migration of unwell migrants (resulting in a lower number of unhealthy migrants in the host society) (Martinez et al., 2015; Mehta & Elo, 2012; Page, 2007; Puschmann et al., 2017). Nevertheless, there continues to be a lack of consensus about the cause of the healthy migrant effect.

Existing studies also indicate that the healthy migrant effect exhibits pronounced differences according to migrants’ countries of origin (Anikeeva et al., 2010; Cunningham et al., 2008; Huijts & Kraaykamp, 2012). For example, while the migrant population in the US has better outcomes in terms of LE, infant mortality and some major causes of death compared with the US-born population, these health advantages are greater for migrants from East Asia, South Asia and Latin America (Cunningham et al., 2008; Mehta et al., 2016; Singh & Miller, 2004). Generally, Asian and Pacific Islander migrants in the US have the highest LE among the foreign-born population (Singh & Miller, 2004). Differences in mortality rates between foreign- and US-born populations and within the foreign-born population are even more significant among elderly people (Mehta et al., 2016) and remain observable within groups of the same ethnicity and race (e.g. US-born and foreign-born Hispanics) (Cho et al., 2004; Cunningham et al., 2008). Variations in health outcomes among different migrant groups according to place of birth have also been observed in many other migrant-receiving countries, including the UK, Belgium (Reus-Pons et al., 2017), the Netherlands (Uitenbroek & Verhoeff, 2002), Australia (Anikeeva et al., 2010; Biddle et al., 2007) and Canada (McDonald & Kennedy, 2004).

### 2.2 Explanations of the effect of nativity on the healthy migrant effect

The striking association between nativity and migrant health disparities is not fully understood. However, it may be partly explained by the characteristics of both the origin and the host countries (Cho et al., 2004; Huijts & Kraaykamp, 2012; Vang et al., 2017).

First, the sociocultural characteristics of migrants’ countries of origin, including the general health of the population, prevailing religious beliefs, traditional dietary practices and political stability, may have consequences for migrant health outcomes (Huijts & Kraaykamp, 2012; Martinez et al., 2015). For example, migrants from countries with a poor level of public health are more likely to experience unfavourable health outcomes compared with those from countries with more developed public health systems (Ho et al., 2007). Likewise, migrants from predominantly Islamic countries have relatively lower rates of mortality compared with other migrants, partly because of the religious focus on temperance and sanctions on unhealthy behaviours such as overindulgence and smoking in females (Huijts & Kraaykamp, 2012). The cultural norms and values as well as socialised health behaviours of countries of origin are important factors contributing to migrant health outcomes (Vang et al., 2017); therefore, the diverse sociocultural backgrounds of migrants may result in health disparities by place of birth.

Second, the characteristics of the host country, particularly the relative size of the established migrant community and the geographic distance from and cultural similarities with the country of origin, are associated with differences in migrant health outcomes (Cunningham et al., 2008; Mehta et al., 2016; Singh & Miller, 2004). Migrants with a higher number of peers from the same country are better able to access social support and have more employment opportunities, thus may have better health outcomes than those with fewer migrant peers (Anson, 2000). For example, Mexican Americans living in communities with a higher concentration of Hispanics have slightly lower rates of mortality than those living in communities with a lower density of Hispanics (LeClere et al., 1997). Additionally, migrants are more likely to be healthy when the host country is geographically distant and has a different culture from that of their country of origin (Martinez et al., 2015). A study of migrant health in 31 European countries found that migrants had better self-rated health status if the geographic distance between the capital cities of their country of origin and host country was greater (Huijts & Kraaykamp, 2012). The reason for this may be that migrants from geographically distant countries or from a country with a different cultural background have had to overcome greater challenges during migration, thus are more selective during the migration process and more likely to be healthy.

### 2.3 Effect of nativity on migrant HLE

Given that the past research on the healthy migrant effect according to nativity has tended to focus either on mortality alone or on mortality and morbidity as separate constructs, there has been a proliferation of research in recent years using HLE as a new dimension to investigate migrants’ health and wellbeing. HLE is a measure of the expected average number of years of healthy life at a given age if current patterns of mortality and morbidity persist (Jagger & Robine, 2011; Stiefel et al., 2010). HLE reflects not only length but also quality of life, thus is a relatively all-encompassing health indicator compared with other health measures (Stiefel et al., 2010). Investigating HLE trends may help improve the understanding of whether an increase in average length of life is associated with a decrease in frailty and disability or an improvement in quality of life (Beltrán-Sánchez et al., 2015). By considering both mortality and morbidity, HLE generates a more integrated understanding of migrant health and its variations by nativity.

The results of existing studies suggest that while migrants have a higher LE, they do not have similar advantages with respect to HLE (Eschbach et al., 2007; Garcia et al., 2017, 2018, 2019; Reus-Pons et al., 2017). For example, compared with US-born Mexican Americans, foreign-born Mexican Americans have a higher LE at 65 years of age. However, their proportion of disability-free LE at age 65 years is lower than that of their US-born counterparts (Eschbach et al., 2007). A study conducted in Belgium found that while migrants from non-Western countries have a higher LE at 50 years of age than that of natives or migrants from Western countries, their HLE is lower (Reus-Pons et al., 2017). Similar findings are reported in Germany (Carnein et al., 2014) and the UK (Wohland et al., 2015).

### 2.4 Factors affecting migrant health outcomes

Migrants' health outcomes are affected by a wide range of pre-migration, migration and post-migration factors. Pre-migration factors affecting migrants' health outcomes are mostly related to the country of origin's socioeconomic development, political stability and cultural practices. For example, migrants from politically repressed countries tend to have long-lasting psychological trauma, while those migrating from countries with poor living conditions and public health systems have lower levels of self-rated health (Huijts & Kraaykamp, 2012). Similarly, Polish migrants who share the similar ethnicity, language and cultural background with Swedish natives have a higher level of self-rated health compared with migrants from Turkey and Iran (Wiking et al., 2004). Hispanic Americans originating from countries that are geographically close to the US (e.g. Mexico or Cuba) have a greater mortality advantage compared with those migrating from Central or South America (Cho et al., 2004). Therefore, we expect that the sociocultural background of the country of origin will have a significant effect on migrants' health outcomes in the host country. Specifically, the health outcomes (e.g. HLE) of European migrants, particularly those from the UK, may be similar to those of Australian-born people given their similar sociocultural background and lower migration selectivity, facilitating their adaptation to Australian society.

During the migration process, the exposure to adverse events, including human trafficking, exploitation, life-threatening events, war and organised violence, may have long-term negative effects on migrants’ wellbeing. For example, a study revealed that Vietnamese refugees in Australia who had experienced three or more types of adverse events during the migration process had a significantly higher risk of mental health disorders 10 years or more after arrival compared with those who had no exposure to adverse events during the migration process (Steel et al., 2002). The self-rated health status of refugees is worse than that of other types of migrants in the US (Jasso et al., 2005) and other settings (Chiswick et al., 2008). Therefore, we hypothesise that migrants who experienced adverse events during the migration process (e.g. Lebanese migrants fleeing war and violence in this study) have a lower LE and HLE compared with other migrant groups.

Post-migration factors affecting migrant health are multifaceted and primarily include behavioural changes, socioeconomic status, language barriers, ethnic enclave residence and discrimination. With a longer duration of residence, migrants are more likely to adopt the dietary and behavioural practices of their host countries. For example, they may adopt unhealthy dietary and lifestyle practices such as the consumption of sweetened drinks and fried food and the use of tobacco, alcohol and drugs, compromising their health and wellbeing over time (Vang et al., 2017). In addition, migrants are typically socioeconomically disadvantaged in their host society and are more likely to suffer from poverty, substandard housing and unemployment (Fennelly, 2007; Myers et al., 1996). For example, they are more likely to encounter difficulties finding employment (including in occupations for which they are qualified) and securing promotions (Fullin & Reyneri, 2011), which are well-established predictors of poor wellbeing (Bartley, 1994). Migrants' wellbeing may also be jeopardised if they experience a language barrier, which limits their access to health and welfare systems (Anikeeva et al., 2010), or migration-related psychological pressures such discrimination, loneliness and social isolation, which are detrimental to their physical and mental wellbeing (Agudelo-Suárez et al., 2011; Farmer et al., 2019). However, living in an ethnic enclave, in which a particular ethnic group is spatially clustered and socioeconomically distinct from the mainstream population, may mitigate the adverse effects of assimilation on migrant health because migrants can more easily maintain their cultural and dietary practices, obtain social support (Portes, 2018) and find employment opportunities (Damm, 2009) within their enclaves. Given the overall adverse effect of acculturation on migrants’ health outcomes, we expect that migrants as a whole may not have a longer HLE compared with the Australian-born population.

The eight migrant groups examined in this study arrived in Australia at different times in history and differ in terms of their original socioeconomic and demographic profiles. We expect considerable variations in LE and HLE outcomes among the eight migrant groups according to socioeconomic status and duration of residence. Specifically, migrants who are socioeconomically advantaged may have better health outcomes compared with those who are socioeconomically disadvantaged. For example, compared with other migrant groups, South African migrants have significantly higher incomes, and Chinese and Indian migrants are better educated (see Table 1). Therefore, we expect these three migrant groups to have a higher LE or HLE compared with other migrants. In addition, British, Italian, Greek and German migrants have an overall longer duration of residence compared with other migrant groups; thus, their health outcomes may converge with the those of Australian-born people. The contingency theory proposes that during the acculturation process, the health status of migrants gradually approaches that of the native-born population (Namer & Razum, 2018). For example, the prevalence of chronic disease among migrants in Australia, despite being lower than that of the Australian-born population, approaches that of the Australian-born population after the first 10 or 20 years of residence (Biddle et al., 2007). Similarly, in Canada, the prevalence of conditions such as asthma, back pain and high blood pressure among migrants from non-English-speaking countries increases more rapidly in the first 20 years following migration before plateauing at similar levels to that of the Canadian-born population (McDonald & Kennedy, 2004). Therefore, we expect that migrant groups with a long duration of residence will have a similar LE and HLE to that of the Australian-born population.

**Table 1:** Sociodemographic characteristics of migrants and their country of birth by nativity in Australia, 2016

	Australian -born	Foreign-born	UK	Germany	Italy	Greece	Lebanon	China	India	South Africa
**Age (%)**										
0-14	24.2	7.5	4.8	2.0	1.0	1.6	2.1	4.0	8.5	7.9
15-24	14.1	10.2	5.0	4.3	1.7	1.8	4.1	21.9	9.7	13.3
25-44	25.0	33.5	18.6	17.6	8.7	5.4	28.8	40.8	60.8	34.4
45-64	23.2	28.7	37.9	25.2	23.1	24.4	44.3	24.1	14.7	32.5
≥65	13.5	20.1	33.6	50.8	65.5	66.8	20.8	9.2	6.3	11.9
**Medium age**	38	44	55	65	70	71	51	33	33	42
**Sex (%)**										
Men	49.5	48.9	50.5	47.1	51.1	47.9	51.8	44.0	53.9	49.2
Women	50.5	51.1	49.5	52.9	48.9	52.1	48.2	56.0	46.1	50.8
**Year of arrival (%)**										
Before 1945	–	0.2	0.4	0.5	0.9	0.4	0.1	0.1	0.0	0.1
1946–1955	–	3.7	6.2	27.7	22.8	10.3	1.4	0.3	0.5	0.2
1956–1965	–	6.9	14.7	18.1	39.4	43.6	4.4	0.8	0.5	1.4
1966–1975	–	10.9	25.1	11.4	20.3	30.8	23.4	0.6	3.6	3.6
1976–1985	–	9.4	10.7	9.5	3.3	4.4	20.6	2.2	2.1	10.1
1986–1995	–	13.4	9.7	6.7	1.7	2.5	21.1	12.8	6.0	11.5
1996–2005	–	16.7	11.8	9.8	2.1	1.8	14.4	20.9	15.7	29.5
After 2006	–	38.8	21.3	16.4	9.5	6.2	14.6	62.1	71.5	43.5
**Median Individual Weekly Income (AU$)**	688	615	709	534	435	392	408	374	785	984
**Participation rate in the labour force (%)**	64.9	58.9	64.6	44.9	31.7	25.4	44.1	52.8	77.3	76.6
**Educational level (%)**										
Postgraduate degree	3.5	9.2	3.9	9.2	2.4	1.3	2.8	17.2	24.7	8.9
Bachelor degree	14.8	21.1	2.8	14.4	5.3	4.5	8.2	25.8	32.2	27.7
Below bachelor	81.8	69.8	93.3	76.4	92.3	94.2	88.9	56.9	43.1	63.4
**Unemployment rate**	6.9	8.4	6.9	5.7	4.3	6.1	8.6	11.8	8.0	6.2
**Index of Relative Socio-economic Advantage and Disadvantage (%)**										
Quintile 1 (most disadvantaged)	18.0	16.7	12.4	15.0	12.9	11.8	27.8	6.5	12.7	5.9
Quintile 2	20.7	15.9	16.6	18.2	17.9	13.2	27.8	7.2	13.6	10.0
Quintile 3	21.2	20.0	21.3	20.5	18.9	16.6	13.3	15.6	23.4	19.2
Quintile 4	19.8	22.9	23.5	20.7	24.2	31.4	19.3	32.6	27.2	26.5
Quintile 5 (most advantaged)	20.3	24.6	26.2	25.7	26.0	26.9	11.8	38.1	23.0	38.3
**GDP per capita of country of origin in 2016 (USD)**	49,875		41,146	42,136	30,960	17,923	8172	8094	1714	5735
**GDP growth rate of country of origin in 2016 (%)**	2.7		2.2	2.2	1.3	−0.5	1.6	6.8	8.3	0.7
**Life expectancy of country of origin in 2016 (years)**	82.5		81.2	81.0	83.2	81.4	79.5	77.2	70.1	64.8

Note: Data were extracted from the Australian Bureau of Statistics (https://www.abs.gov.au/), Depart of Home Affairs of Australian Government (https://www.homeaffairs.gov.au/about-us/our-portfolios/multicultural-affairs/community-information-summaries/country-list-of-summaries) and World Bank (https://datatopics.worldbank.org/world-development-indicators/).

Moreover, the health disparities among different migrant groups may decrease with increasing age according to the theory of age-as-leveler, which posits the existence of a biological ceiling, meaning that people become universally fragile as they age, despite their different sociocultural characteristics, which become less important over time (Dupre, 2007; Ferraro & Farmer, 1996). The expected reduction in health disparities according to nativity is also attributable to mortality selection in which individuals from socioeconomically disadvantaged groups are more likely to die prematurely because of poor health compared with those in socioeconomically advantaged groups (Beckett, 2000). Therefore, we expect that the disparities in LE and HLE between different migrant groups may diminish with increasing age.

## 3 Data and methods

### 3.1 Data

We draw on data from the Australian Bureau of Statistics (ABS) to construct life tables for both the Australian-born population and migrants born in eight foreign countries (the UK, Germany, Greece, Italy, Lebanon, China, India and South Africa) for 2006, 2011 and 2016. Specifically, the size of the total Australian population is derived from the ABS's annual estimated resident population, while the population sizes of Australian-born individuals and migrants born in the eight foreign countries are estimated from 1% of data randomly selected from the Census of Population and Housing (CPH) (2006: N = 199,406; 2011: N = 215,597; 2016: N = 235,408), adjusted by the ratio of the estimated resident population to the CPH population. The number of deaths by country of birth is obtained from annual mortality statistics published by the ABS, while information about migrant health status by country of birth is collected from the 1% of data randomly selected from the CPH. All above data are categorised into five age groups: 45–54, 55–64, 65–74, 75–84 and 85+ years. Data for people under the age of 45 years are excluded because mortality data are incomplete for migrants of this age group originating from certain countries in certain years. Life tables are constructed using the method proposed by Chiang (1979).

### 3.2 Health measures

The CPH measures health according to one's ability to independently perform three core activities: self-care, mobility and communication. Self-care is defined as the ability to perform daily activities of living such as eating, showering, dressing or toileting; mobility pertains to the ability to move, such as getting out of bed and moving around the home or outside of the home; and communication is defined as the ability to understand or be understood by others. If respondents require assistance with any of these core activities because of disability, a chronic health condition (lasting longer than 6 months) or old age, they are considered unhealthy. In contrast, communication difficulties because of a lack of proficiency in the English language are not considered an indicator of poor health. Respondents who experience no difficulty in performing any of the three core activities are considered healthy. The inclusion of communication in the health measure improves the evaluation of an individual's care needs, which are crucially influenced by the care recipient's communication ability, which in turn is an important indicator of health status (Threats & Worrall, 2004). In this study, communication ability is measured by an individual's capacity to understand or be understood by others without the need for help or supervision arising from a disability, long-term illness or old age.

### 3.3 Methods

To estimate HLE, we use the Sullivan method (Sullivan, 1971), which is widely used in the HLE research because it is based on highly available cross-sectional data and does not involve complex computational procedures, thus is operationally feasible (Mathers et al., 2001). This differs from other HLE estimation approaches such as the multistate life table method, which requires longitudinal data and a large number of computations. The procedures of the Sullivan method can be found in detail elsewhere (Jagger et al., 2014) and are briefly summarised below.

Equations (1), (2) illustrate the computation of LE and HLE in this study based on the Sullivan method:

(1) LEx=∑xωLxnlx

(2) HLEx=∑xω(1−πxn)*Lxnlx

where *x* represents age, ω denotes the highest age, and *n* denotes the age group interval; $l_{x}$ represents the number of persons surviving to age *x*; $LE_{x}$ and $HLE_{x}$ denote LE and HLE, respectively, at age *x*; πxn is the proportion of unhealthy people in the age group (*x, x* + *n*); and Lxn corresponds to the number of person years lived in the age group (*x, x* + *n*) based on the conventional life table method. Computations for $l_{x}$ and Lxn can be found in Chiang (1979). Estimation of the confidence interval (CI) of LE is based on the method proposed by Chiang (1979), while the estimation of the CI of HLE is based on the method proposed by Jagger et al. (2014). In addition to LE and HLE, we also estimate the HLE/LE ratio, which denotes the proportion of remaining healthy life at a given age. A high HLE/LE ratio indicates a high quality of life, particularly with an increase in LE, because individuals are more likely to be healthy while living longer.

## 4 Results

### 4.1 Prevalence of crude death rate and unhealthy status by country of birth

Table 2 presents the age-specific and age-standardised crude death rates and unhealthy status of the Australian- and foreign-born populations. The differences in crude death rate and unhealthy status according to nativity are notable. Following age standardisation (using the Australian-born population in 2006 as the standard population), the foreign-born population overall had a lower mortality rate, but a higher level of poor health, compared with the Australian-born population. For example, in 2016, the age-standardised death rate of the foreign-born population older than 45 years was 13.2 per 1000 persons, which was lower than that of Australian-born individuals of the same age (15.4 per 1000 persons). Conversely, the age-standardised proportion of poor health for migrants older than 45 years in the same year was 10.6%, which was higher than that of Australian-born individuals older than 45 years (8.8%).

**Table 2:** Crude death rate and proportion of unhealthy status of australians by age group and country of birth: 2006, 2011 and 2016.

Age group	Australian -born	Foreign-born	UK	Germany	Italy	Greece	Lebanon	China	India	South Africa
		Crude Death Rate (per 1000 persons)								
	**2006**									
45–54	2.6	1.9	2.1	2.8	1.9	2.8	1.9	1.2	0.8	1.3
55–64	5.7	4.9	5.3	6.5	4.5	4.2	5.4	3.8	3.9	3.5
65–74	15.4	13.1	13.9	14.8	11.7	10.7	14.5	10.5	11.8	9.6
75–84	45.5	41.3	45.6	41.7	38.1	35.9	48.2	35.1	41.8	36.8
≥85	144.0	126.1	137.9	114.8	130.7	129.1	105.5	121.3	128.2	126.3
Total^a^	17.1	14.9	16.3	15.6	14.4	14.1	15.2	12.9	14.1	13.2
	**2011**									
45–54	2.6	1.7	2.0	3.1	1.9	1.7	1.5	1.1	0.9	0.8
55–64	5.5	4.7	5.1	7.0	4.7	4.8	4.9	2.9	3.1	3.2
65–74	13.5	12.3	13.2	12.8	12.6	10.0	13.2	8.0	9.6	11.2
75–84	42.5	37.6	40.4	40.9	36.1	32.8	43.2	24.8	29.4	27.4
≥85	143.9	126.1	140.5	119.6	115.4	112.1	124.0	100.1	116.5	120.3
Total^a^	16.3	14.2	15.5	15.6	13.7	12.6	14.9	10.0	11.6	11.7
	**2016**									
45–54	2.5	1.7	2.0	1.6	2.0	1.7	1.9	0.9	0.8	1.0
55–64	5.4	4.1	4.8	5.7	3.6	4.6	5.0	1.8	3.4	3.0
65–74	12.4	10.9	12.2	12.6	10.2	9.8	13.4	5.9	9.2	8.4
75–84	38.7	34.0	37.1	37.2	33.0	31.9	43.2	23.7	29.4	25.0
≥85	137.9	123.9	145.2	127.5	116.3	108.6	121.5	92.5	108.4	115.0
Total^a^	15.4	13.2	15.1	14.5	12.6	12.3	15.0	8.8	11.2	10.8
		Proportion of Unhealthy Status (%)								
	**2006**									
45–54	3.1	2.7	2.4	2.6	2.7	3.8	7.4	1.7	1.4	1.0
55–64	5.2	5.3	4.3	4.6	5.4	8.1	13.4	4.5	3.7	2.2
65–74	7.5	9.4	6.5	6.4	11.5	13.4	20.2	12.3	8.6	5.1
75–84	20.5	24.7	18.9	20.8	30.8	33.5	42.5	30.4	23.2	16.9
≥85	53.0	53.1	50.6	53.5	66.0	68.1	62.8	62.3	57.9	48.6
Total^a^	8.8	9.5	7.8	8.3	11.2	13.1	18.0	10.5	8.4	6.1
	**2011**									
45–54	3.5	3.0	2.5	2.5	3.1	4.1	10.2	1.7	1.5	1.1
55–64	5.6	6.0	4.6	5.5	5.6	9.0	17.8	5.2	4.3	2.3
65–74	8.3	10.5	7.3	7.4	12.6	15.8	26.0	12.7	9.1	5.1
75–84	20.0	26.1	18.4	20.7	32.8	36.1	48.7	33.0	25.3	16.6
≥85	51.8	54.4	49.6	52.8	64.5	67.8	71.6	65.3	55.9	50.1
Total^a^	9.0	10.2	7.9	8.6	11.8	14.2	22.5	11.2	8.9	6.2
	**2016**									
45–54	4.0	3.3	2.6	2.6	3.6	4.5	9.9	1.5	1.8	1.3
55–64	5.9	6.4	4.9	5.6	6.1	8.4	18.8	4.9	4.8	2.8
65–74	9.0	10.9	7.8	7.9	11.3	17.0	28.1	11.5	10.4	5.8
75–84	19.7	26.1	18.3	18.8	32.7	36.2	47.5	35.7	27.0	16.4
≥85	50.9	55.6	49.9	51.4	64.6	67.8	73.9	68.1	55.5	49.4
Total^a^	9.4	10.6	8.1	8.5	11.9	14.4	22.9	11.3	9.6	6.4

a Age standardised using the Australian-born population in 2006 as the standard population.

Importantly, age-standardised death rate and poor health status varied significantly by country of birth among the foreign-born population. Specifically, Chinese migrants over the age of 45 years had the lowest age-standardised death rate (8.8–12.9 per 1000 persons between 2006 and 2016), followed by migrants from India and South Africa (10.8–14.1 per 1000 persons), while those from the UK, Germany and Lebanon suffered a relatively higher age-standardised death rate (14.5–16.3 per 1000 persons). With respect to the age-standardised proportion of poor health, South Africans over the age of 45 years experienced the lowest rate of poor health (6.1–6.4% between 2006 and 2016), followed by British and German migrants (7.8–8.6%), while other migrant groups had relatively higher figures, particularly those from Lebanon (18.0–22.9%). Such differences according to nativity are also found for age-specific crude death rates and age-specific proportion of poor health across all three years of the study.

### 4.2 Variations in LE by country of birth

**Fig. 3:**
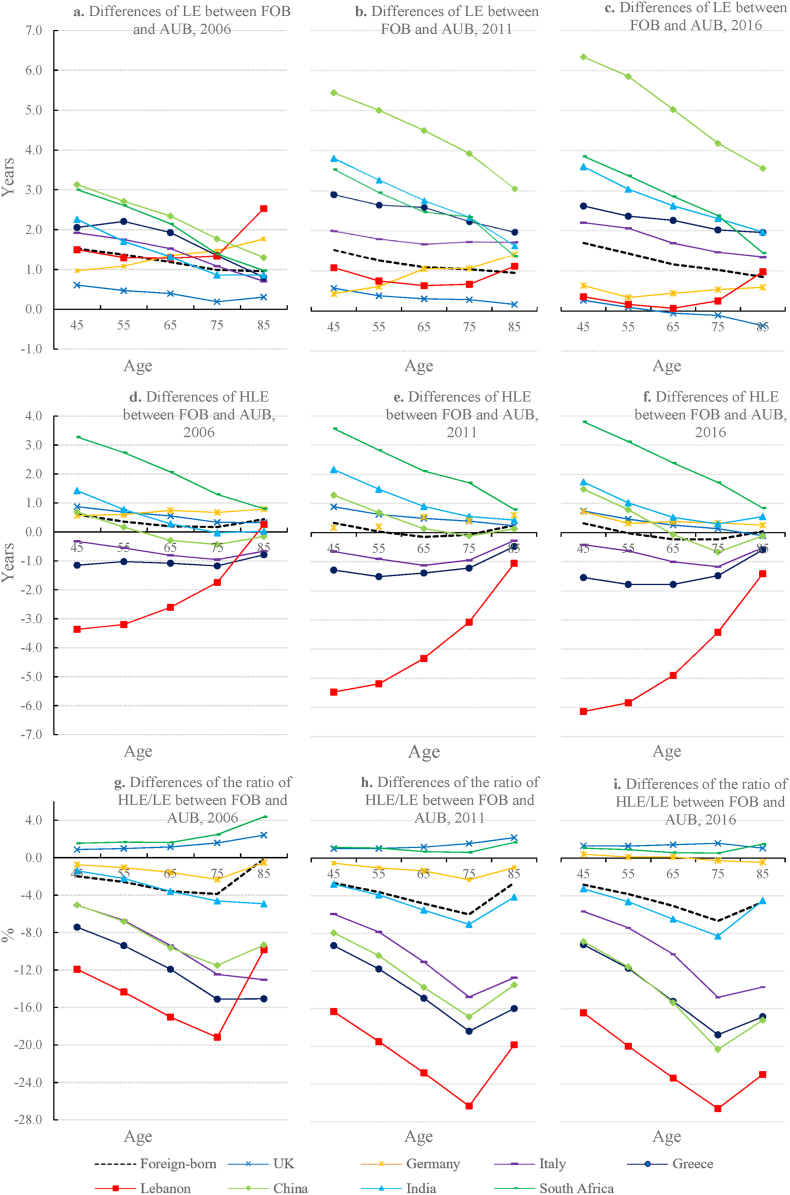
Differences Between LE, HLE and HLE/LE Ratio for Australian-Born and Eight Foreign-Born Populations Note: 1. AUB: Australian-born; FOB: foreign-born. 2. The lines in the sub-figures (a), (b) and (c) describe to what extent the LE of the foreign-born population exceeded that of the Australia-born population in 2006, 2011 and 2016, respectively. For example, in sub-figure (a), for x = 45 and y = 1.5, it means that LE of the foreign-born population exceeded that of the Australian by 1.5 years at age 45 years in 2006. Likewise, for the sub-figures (d), (e) and (f) regarding the differences of HLE between FOB and AUB, as well as the sub-figures (g), (h) and (i) regarding the differences of the ratio HLE/LE between FOB and AUB, they can be interpreted in the same way. 3. The first widened but then reduced nativity differences of the HLE/LE ratio can be explained by the different extents of reduction in nativity differences regarding LE and HLE as age increases. Before age 85, the decrease in nativity differences of HLE (i.e., nominator) (see Fig. 3d-f) is relatively small than that for nativity differences of LE (i.e., denominator) (see Fig. 3a-c); this, therefore, leads to enlarging nativity differences of the HLE/LE ratio. However, at age 85 years and older, the decrease in nativity differences of HLE is relatively large than that for nativity differences of LE, therefore, reducing the nativity difference in the HLE/LE at the 85+ years.

Table 3 and Fig. 2, Fig. 3 show the LE, HLE and HLE/LE ratio at five ages—45, 55, 65, 75 and 85years—in the Australian-born population and the eight major migrant groups in 2006, 2011 and 2016. Notably, LE, HLE and HLE/LE ratio varied significantly according to nativity.

**Table 3:** Life expectancy (LE), healthy life expectancy (HLE), and HLE/LE ratio by age and country of birth in Australia: 2006, 2011 and 2016.

	2006			2011			2016		
	LE (years)	HLE (years)	HLE/LE (%)	LE (years)	HLE (years)	HLE/LE (%)	LE (years)	HLE (years)	HLE/LE (%)
Australian-born									
45	37.8(37.8,37.8)	33.2(33.2,33.2)	87.9	38.3(38.3,38.3)	33.5(33.4,33.5)	87.4	38.9(38.9,38.9)	33.8(33.7,33.8)	86.8
55	28.6(28.6,28.6)	24.2(24.2,24.3)	84.7	29.1(29.1,29.2)	24.6(24.5,24.6)	84.3	29.8(29.8,29.8)	24.9(24.9,25.0)	83.7
65	20.0(20.0,20.0)	15.9(15.9,15.9)	79.5	20.5(20.5,20.5)	16.2(16.2,16.3)	79.2	21.1(21.1,21.1)	16.6(16.6,16.7)	78.7
75	12.5(12.5,12.5)	8.5(8.5,8.6)	68.2	12.8(12.7,12.8)	8.8(8.8,8.8)	68.8	13.3(13.3,13.3)	9.1(9.1,9.2)	68.8
85	6.9(6.9,7.0)	3.3(3.2,3.3)	47.0	6.9(6.9,7.0)	3.4(3.3,3.4)	48.2	7.3(7.2,7.3)	3.6(3.5,3.6)	49.1
**Foreign-born**									
45	39.3(39.3,39.3)	33.8(33.7,33.8)	85.9	39.3(39.3,39.3)	33.8(33.7,33.8)	84.8	40.6(40.6,40.6)	34.1(34.0,34.1)	84.0
55	30.0(29.9,30.0)	24.6(24.5,24.6)	82.1	30.0(29.9,30.0)	24.6(24.5,24.6)	80.7	31.2(31.2,31.2)	24.9(24.9,24.9)	79.9
65	21.2(21.2,21.3)	16.1(16.1,16.2)	75.9	21.2(21.2,21.3)	16.1(16.1,16.1)	74.4	22.3(22.3,22.3)	16.4(16.4,16.4)	73.6
75	13.5(13.5,13.5)	8.7(8.7,8.7)	64.3	13.5(13.5,13.5)	8.7(8.7,8.7)	62.8	14.3(14.3,14.3)	8.9(8.8,8.9)	62.1
85	7.9(7.9,8.0)	3.7(3.7,3.8)	46.9	7.9(7.9,8.0)	3.6(3.6,3.7)	45.6	8.1(8.0,8.1)	3.6(3.6,3.7)	44.4
**UK**									
45	38.4(38.4,38.4)	34.1(34.0,34.2)	88.8	38.8(38.8,38.9)	34.4(34.2,34.5)	88.4	39.2(39.1,39.2)	34.5(34.4,34.6)	88.1
55	29.1(29.1,29.1)	24.9(24.8,25.0)	85.7	29.5(29.5,29.6)	25.2(25.1,25.3)	85.3	29.9(29.8,29.9)	25.4(25.3,25.5)	85.0
65	20.4(20.4,20.4)	16.5(16.4,16.5)	80.6	20.8(20.8,20.8)	16.7(16.7,16.8)	80.4	21.1(21.1,21.1)	16.9(16.8,17.0)	80.1
75	12.7(12.7,12.7)	8.9(8.8,8.9)	69.7	13.0(13.0,13.1)	9.2(9.1,9.2)	70.3	13.2(13.1,13.2)	9.3(9.2,9.3)	70.3
85	7.3(7.1,7.5)	3.6(3.5,3.7)	49.4	7.1(7.0,7.3)	3.6(3.5,3.6)	50.4	6.9(6.7,7.1)	3.5(3.4,3.5)	50.1
**Germany**									
45	38.7(38.6,38.9)	33.8(33.4,34.1)	87.1	38.7(38.6,38.8)	33.6(33.3,34.0)	86.9	39.5(39.4,39.6)	34.5(34.1,34.9)	87.2
55	29.7(29.6,29.8)	24.8(24.6,25.1)	83.6	29.8(29.7,29.8)	24.8(24.5,25.1)	83.3	30.1(30.0,30.2)	25.2(24.9,25.6)	83.8
65	21.4(21.3,21.5)	16.6(16.4,16.9)	77.9	21.6(21.5,21.6)	16.8(16.6,17.0)	77.9	21.6(21.5,21.7)	17.0(16.8,17.2)	78.8
75	14.0(13.9,14.1)	9.2(9.0,9.4)	65.9	13.8(13.7,13.9)	9.2(9.0,9.4)	66.5	13.8(13.7,13.9)	9.5(9.3,9.6)	68.5
85	8.7(8.6,8.8)	4.0(3.9,4.2)	46.5	8.4(8.3,8.4)	3.9(3.8,4.1)	47.2	7.8(7.8,7.9)	3.8(3.6,4.0)	48.6
**Italy**									
45	39.7(39.6,39.8)	32.9(32.7,33.1)	82.8	40.3(40.2,40.3)	32.8(32.6,33.0)	81.5	41.1(41.0,41.2)	33.4(33.1,33.6)	81.2
55	30.4(30.3,30.4)	23.7(23.5,23.8)	78.0	30.9(30.9,31.0)	23.7(23.5,23.8)	76.5	31.8(31.8,31.9)	24.3(24.1,24.5)	76.3
65	21.5(21.5,21.6)	15.1(15.0,15.2)	70.1	22.2(22.1,22.2)	15.1(15.0,15.2)	68.2	22.8(22.8,22.9)	15.6(15.5,15.7)	68.5
75	13.6(13.6,13.6)	7.6(7.5,7.7)	55.7	14.5(14.4,14.5)	7.8(7.8,7.9)	54.0	14.7(14.7,14.8)	8.0(7.9,8.0)	54.0
85	7.6(7.6,7.7)	2.6(2.5,2.7)	34.0	8.7(8.6,8.7)	3.1(3.0,3.2)	35.5	8.6(8.6,8.6)	3.0(3.0,3.1)	35.4
**Greece**									
45	39.8(39.7,39.9)	32.1(31.8,32.3)	80.5	41.2(41.1,41.3)	32.2(31.9,32.5)	78.1	41.5(41.4,41.6)	32.2(31.9,32.6)	77.6
55	30.8(30.8,30.9)	23.2(23.0,23.4)	75.3	31.8(31.7,31.9)	23.1(22.8,23.3)	72.5	32.1(32.1,32.2)	23.1(22.9,23.4)	72.0
65	21.9(21.9,22.0)	14.8(14.7,15.0)	67.6	23.1(23.0,23.2)	14.9(14.7,15.0)	64.3	23.4(23.3,23.5)	14.9(14.7,15.0)	63.5
75	13.9(13.8,13.9)	7.4(7.2,7.5)	53.1	15.0(14.9,15.1)	7.6(7.5,7.7)	50.4	15.3(15.2,15.4)	7.7(7.6,7.7)	50.0
85	7.7(7.7,7.8)	2.5(2.4,2.6)	31.9	8.9(8.9,9.0)	2.9(2.8,3.0)	32.2	9.2(9.2,9.3)	3.0(2.9,3.1)	32.2
**Lebanon**									
45	39.3(39.1,39.5)	29.8(29.4,30.3)	76.0	39.4(39.2,39.5)	28.0(27.7,28.3)	71.1	39.3(39.1,39.4)	27.6(27.3,27.9)	70.4
55	29.9(29.7,30.1)	21.0(20.6,21.5)	70.3	29.9(29.7,30.1)	19.4(19.1,19.7)	64.8	29.9(29.8,30.1)	19.1(18.8,19.4)	63.7
65	21.3(21.1,21.5)	13.3(12.9,13.7)	62.5	21.1(21.0,21.3)	11.9(11.7,12.2)	56.4	21.2(21.1,21.4)	11.7(11.5,12.0)	55.3
75	13.8(13.7,14.0)	6.8(6.5,7.1)	49.0	13.4(13.3,13.6)	5.7(5.5,5.9)	42.5	13.5(13.4,13.7)	5.7(5.5,5.9)	42.2
85	9.5(9.2,9.7)	3.5(3.2,3.8)	37.2	8.1(7.9,8.3)	2.3(2.1,2.5)	28.4	8.2(8.0,8.4)	2.1(2.0,2.3)	26.1
**China**									
45	40.9(40.8,41.0)	33.9(33.6,34.2)	82.8	43.7(43.6,43.8)	34.8(34.5,35.0)	79.5	45.2(45.2,45.3)	35.3(35.1,35.5)	78.0
55	31.3(31.2,31.5)	24.4(24.1,24.7)	77.9	34.1(34.0,34.2)	25.3(25.0,25.5)	74.0	35.6(35.5,35.7)	25.7(25.5,25.9)	72.2
65	22.4(22.2,22.5)	15.6(15.4,15.9)	69.9	25.0(24.9,25.1)	16.4(16.2,16.6)	65.5	26.2(26.1,26.2)	16.6(16.4,16.7)	63.3
75	14.3(14.2,14.4)	8.1(7.9,8.3)	56.7	16.7(16.6,16.8)	8.7(8.5,8.8)	51.9	17.5(17.4,17.5)	8.5(8.3,8.6)	48.5
85	8.2(8.1,8.3)	3.1(2.9,3.3)	37.7	10.0(9.9,10.1)	3.5(3.3,3.6)	34.7	10.8(10.7,10.9)	3.4(3.3,3.6)	31.9
**India**									
45	40.0(39.9,40.2)	34.6(34.2,35.0)	86.5	42.1(42.0,42.2)	35.6(35.3,36.0)	84.7	42.5(42.4,42.6)	35.5(35.2,35.8)	83.6
55	30.3(30.2,30.5)	25.0(24.6,25.4)	82.5	32.4(32.3,32.5)	26.1(25.7,26.4)	80.4	32.8(32.7,32.9)	25.9(25.7,26.2)	79.1
65	21.3(21.2,21.5)	16.2(15.8,16.5)	75.9	23.3(23.1,23.4)	17.1(16.9,17.4)	73.7	23.8(23.7,23.9)	17.2(16.9,17.4)	72.2
75	13.4(13.2,13.5)	8.5(8.2,8.8)	63.6	15.1(15.0,15.2)	9.3(9.1,9.6)	61.7	15.6(15.5,15.7)	9.4(9.2,9.6)	60.5
85	7.8(7.6,7.9)	3.3(3.0,3.5)	42.1	8.6(8.5,8.7)	3.8(3.6,4.0)	44.1	9.2(9.1,9.3)	4.1(3.9,4.3)	44.5
**South Africa**									
45	40.8(40.6,41.0)	36.5(35.9,37.0)	89.4	41.8(41.7,42.0)	37.0(36.6,37.5)	88.6	42.8(42.6,42.9)	37.6(37.2,38.0)	87.9
55	31.2(31.0,31.4)	27.0(26.4,27.5)	86.4	32.1(31.9,32.2)	27.4(27.0,27.8)	85.4	33.1(33.0,33.3)	28.0(27.7,28.4)	84.6
65	22.2(22.0,22.4)	18.0(17.5,18.5)	81.1	23.0(22.8,23.1)	18.4(17.9,18.8)	79.9	24.0(23.9,24.1)	19.0(18.7,19.4)	79.3
75	13.9(13.7,14.1)	9.8(9.4,10.2)	70.7	15.1(15.0,15.3)	10.5(10.2,10.8)	69.4	15.7(15.5,15.8)	10.8(10.6,11.1)	69.3
85	7.9(7.7,8.0)	4.1(3.7,4.5)	51.4	8.3(8.2,8.4)	4.1(3.8,4.5)	49.9	8.7(8.6,8.8)	4.4(4.1,4.7)	50.6

Note: 95% confidence interval is presented in parentheses.

Overall, all eight migrant groups (i.e. from the UK, Germany, Italy, Greece, Lebanon, India, China and South Africa) had a higher LE than that of the Australian-born population (see Table 3 and Fig. 2a). For example, in 2016, the LE of migrants at age 45 years ranged from 39.2 years (95% CI [39.1, 39.2]) to 45.2 years (95% CI [45.2, 45.3]), with an average of 40.6 years (95% CI [40.6, 40.6]), which was well above that of Australian-born individuals at the same age (38.9 years, 95% CI [38.9, 38.9]). Migrants from China, India and South Africa had the highest LE among the eight migrant groups. Compared with the Australian-born population, Chinese migrants had the highest LE (a difference of 3.1 years in 2006, 5.4 years in 2011 and 6.3 years in 2016), while Indian and South African migrants also had a significantly higher LE, although the difference was smaller (2.3–3.9 years). The difference in LE at age 45 years for Italian and Greek migrants (1.9–2.9 years) was not as large as that for Chinese, Indian and South African migrants, while British and German migrants only had a slightly higher LE at age 45 compared with the Australian-born population (a difference of 0.4–1 years) (see Fig. 2a and 3a–3c).

The LE trends from 2006 to 2016 also differed between the eight migrant groups. While LE at age 45 years for migrants from China, India, South Africa, Italy and Greece exceeded that of the Australian-born population by an increasing margin (0.3–3.2 years) in 2006, 2011 and 2016, the gap in LE between the Australian-born population and the other three migrant groups (i.e. those from the UK, Germany and Lebanon) experienced a reverse trend, decreasing by 0.3–1.1 years over the same period. This decreasing trend was particularly prominent among Lebanese migrants, whose LE at age 45 years remained almost unchanged over the 10-year study period. Consequently, the LE of Lebanese migrants at age 45 years exceeded that of the Australian-born population by 1.5 years in 2006 but only by 0.4 years in 2016 (see Fig. 2a and 3a–3c).

### 4.3 Variations in HLE and HLE/LE ratio by nativity

Although all migrant groups had a higher LE at age 45 years and above, their HLE was only slightly higher than that of Australian-born individuals of the same age. Additionally, the HLE/LE ratio of many migrant groups was even lower than that of the Australian-born population. Specifically, while migrants born in five foreign countries (South Africa, India, China, the UK and Germany) could expect a higher HLE at age 45 years compared with that of the Australian-born population, those originating from the other three foreign countries (Italy, Greece and Lebanon) suffered a lower HLE at age 45 years (by 0.3–3.4, 0.6–5.5 and 0.4–6.1 years in 2006, 2011 and 2016, respectively) (see Fig. 2b and 3d–3f). With respect to the HLE/LE ratio, the foreign-born population as a whole and most migrant groups had a lower HLE/LE ratio than that of the Australian-born population. Specifically, migrants at age 45 years were expected to remain healthy for 85.9%, 84.8% and 84.0% of their remaining life in 2006, 2011 and 2016, respectively, which was lower than that of their Australian-born counterparts at the same age (87.9%, 87.4% and 86.8%, respectively). Only migrants from the UK, Germany and South Africa had a moderately higher or similar HLE/LE ratio to that of the Australian-born population, while those born in India, China, Italy and Greece exclusively showed a significantly lower HLE/LE ratio. The HLE/LE ratio at age 45 years of Lebanese migrants was especially low compared with that of the Australian-born population (76.0% v. 87.9%, 71.1% v. 87.4% and 70.4% v. 86.8% in 2006, 2011 and 2016, respectively) (see Fig. 2c). Such differences in the HLE/LE ratio between the Australian-born individuals and all eight migrant groups, particularly those from China, Italy, Greece and Lebanon, remained significant for all ages above 45 years (see Fig. 3g–i).

### 4.4 Variations in LE, HLE and the HLE/LE ratio by nativity, age group and year

The differences in LE, HLE and HLE/LE ratio between migrants and Australian-born individuals show different age patterns. As shown in Fig. 3a, b and 3c, the differences in LE by nativity generally narrowed as age increased, with the smallest differences being at age 85 years. An exception was the difference in LE between Lebanese migrants and the Australian-born population, which, despite gradually decreasing in those under the age of 85 years, spiked at age 85 years. With respect to the differences in HLE by nativity (see Fig. 3e, e and 3f), as age increased, the corresponding values approached to approximately zero; therefore, the differences in HLE according to nativity gradually disappeared with ageing. This was observed for migrant groups with either a higher or a lower HLE compared with Australian-born individuals. Given the convergence in HLE of migrants and Australian-born individuals, the difference in the HLE/LE ratio narrowed at age 85 years for Chinese, Lebanese, Italian and Greek migrants, whose HLE was lower than that of the Australian-born population under the age of 85 years, before converging to that of the Australian-born population at 85+ years.

The differences in LE, HLE and HLE/LE ratio between migrants and Australian-born individuals did not vary significantly in 2006, 2011 and 2016 (see Fig. 3). However, Lebanese migrants exhibited a primarily downward trend in all three indicators, implying worse health outcomes for this migrant group. The LE of Chinese and Indian migrants increased at a faster rate than that of Australian-born individuals from 2006 to 2016, although their HLE and HLE/LE ratio were similar to those of Australian-born individuals in all three years.

## 5 Discussion and conclusion

### 5.1 Summary of results

An understanding of migrant health, particularly variations in health outcomes among different migrant groups, is crucial for the allocation of healthcare resources and targeting vulnerable groups. Nevertheless, prior to this study, research on migrant HLE by nativity was lacking, particularly in the context of Australia. This study provides the first exploration of migrant HLE by nativity in Australia, showing that, on average, the migrant population had a significantly higher LE, a slightly higher HLE and a lower HLE/LE ratio than that of the Australian-born population. Remarkable differences in migrant health outcomes according to country of birth were also observed. Specifically, compared with the Australian-born population, South African, British and German migrants had a higher or similar LE, HLE and HLE/LE ratio. In contrast, Chinese, Indian, Italian and Greek migrants had a significantly higher LE, but a lower HLE/LE ratio, compared with their Australian-born counterparts. Notably, among the eight migrant groups, Lebanese migrants experienced the poorest health outcomes, being the only migrant group showing a declining HLE and HLE/LE ratio from 2006 to 2016. These results offer a solid, evidence-based understanding of the disparities in health and the healthy migrant effect, as measured by LE and HLE, with respect to age, year and country of birth among different migrant groups in Australia. These findings imply that migrants do not have the same advantages with respect to morbidity as they do for mortality and that there are significant variations and patterns in both mortality and morbidity outcomes according to nativity.

### 5.2 Healthy migrant effect for migrants as a whole

The results support the claim that the healthy migrant effect pertains to mortality but not to morbidity; that is, although migrants may have a longer life, they do not necessarily have a healthier life. This finding may be plausibly explained by a number of factors, including migration selectivity, the ‘salmon effect’, statistical bias and convergence theory.

Migration selectivity, in which healthier people are more likely than unhealthy people to successfully migrate and settle in their destination country, may partially explain the higher LE of migrants (Anderson et al., 2004; Cho et al., 2004; Mehta & Elo, 2012). Migration selectivity is also a result of a host country's immigration policies, which generally mandate minimum health requirements to safeguard the country's healthcare system. Migrants' mortality advantage may be influenced by both the protective effect of the original culture, in which the dietary practices and sedentary lifestyle related to increased morbidity in industrialised societies have not been adopted, and the immediate benefits from advanced healthcare systems of the host society (Razum & Twardella, 2002; Young & Hopkins, 2014). This is particularly significant for migrants living in ethnic enclaves, where they are more likely to maintain their traditional dietary practices, build sociocultural connections and find employment opportunities (Eschbach et al., 2004; Moore, 1989). However, migrants' better mortality outcomes could be a data artefact caused by reporting bias (e.g. age misreporting and misclassification of nationality/ethnicity) (Smith & Bradshaw, 2006; Uitenbroek & Verhoeff, 2002) or return migration. The latter is referred to as the salmon effect (Abraido-Lanza et al., 1999; Palloni & Arias, 2004), which is the propensity of migrants to return to their country of origin when they become unhealthy, unemployed or old, artificially reducing the number of unhealthy migrants in the host society (Shai & Rosenwaike, 1987).

The similar and even slightly lower HLE of migrants found in this study demonstrates that migrants' better health status may be compromised by their exposure to hardships and negative experiences in the pre- and post-migration processes. The migration process is generally stressful, characterised by lengthy visa applications, comprehensive file preparations and anxiety arising from uncertainties (Bhugra, 2004). For illegal migrants or refugees, the migration process may even be life-threatening because of human trafficking, war and organised violence, which may elicit long-lasting psychological trauma (Bogic et al., 2015). The post-migration environment may also be detrimental to migrants' health, particularly for those with better health outcomes on arrival. This is illustrated by the so-called contingency theory (Namer & Razum, 2018), which holds that during the assimilation and integration process, migrants gradually adopt the health behaviours of the destination country; consequently, migrants' health status converges with that of the native-born population (Namer & Razum, 2018). For example, migrants may adopt unhealthy behaviours such as smoking, alcohol consumption and high-sugar, high-fat diets during acculturation, leading to deteriorating health (Pierce et al., 2007). Other migration-related difficulties during the assimilation process such as discrimination, loneliness, social isolation and language barriers when accessing medical resources may also compromise migrant health and wellbeing in the long term (Agudelo-Suárez et al., 2011). These factors, although not life-threatening, may diminish migrants’ morbidity advantages over time, reflected by the relatively higher LE but lower HLE observed in this study for migrants in general.

Migrants' lower HLE/LE ratio indicates that when both mortality and morbidity are considered, migrants may experience a lower quality of life compared with the Australian-born population. That is, the healthy migrant effect may not be valid if different aspects of health are considered. This is particularly true for some migrants (e.g. Chinese and Indian) examined in this study, who had a significantly longer LE, but a lower ratio, compared with the Australian-born population. This may be explained by migrants' exposure to migration- and acculturation-related hardships, which erode migrants' health advantages over time. It also suggests that migrants, despite playing an increasing role in the society and economy of many migrant-receiving countries, may not be sufficiently supported by the healthcare systems in their host societies. Migrants may lack the so-called navigational capital needed to access and utilise healthcare resources, despite having a higher income than that of the native population (Yosso, 2005). This has particular caveats for Australia in that migrants’ faster health deterioration over time implies that there may be considerable pressure on the Australian healthcare system with the increase in the migrant population. Thus, policymakers should focus on improving healthcare support and resources for migrants, particularly those from different culture and linguistic backgrounds and those who are unfamiliar with the Australian healthcare system, hindering their access to healthcare resources.

### 5.3 Differences in the healthy migrant effect by nativity

This study also reveals the considerable health disparities related to both mortality and morbidity among different Australian migrant groups. The phenomenon that migrants from China, India, Italy, Greece and Lebanon have a longer LE but a significantly lower HLE/LE ratio compared with the Australian-born population is particularly intriguing. This finding offers a detailed insight into some of the important aspects of the healthy migrant effect and migrant health profiles, especially their changes by age, year and country of birth in the Australian context. The results suggest that when considering the healthy migrant effect among migrant groups, public health policymakers and aged care service providers should consider the quality of life and disparities in aged care needs among ageing populations. Understanding migrants’ cultural backgrounds, duration of residence, migration experiences and socioeconomic status may be useful in interpreting the considerable differences observed for LE and HLE according to place of birth.

First, a close cultural connection between the original and host society may contribute to migrants' reduced mortality advantage and enhanced morbidity advantage. Compared with those from countries with a vastly different cultural background, migrants from countries that are culturally similar to the host country face fewer difficulties during the migration process, particularly with respect to language, meaning that they are less selective, thus show a reduced healthy migrant effect with respect to mortality. For example, migrants in the US from culturally similar countries or regions such as Northern Europe, Oceania and Canada have a lower mortality advantage compared with those from South Central Asia and Eastern Asia, where the sociocultural profile differs from that of the US (Wohland et al., 2015). However, compared with those from countries with a different cultural background, migrants from culturally similar countries may have more advantages during the acculturation process in their post-migration life, including building a social network (du Plooy et al., 2020), finding employment (Kaas & Manger, 2012) and being less likely to experience discrimination (Kingston et al., 2015). Previous studies have demonstrated that advantages such as greater social capital (Lecerof et al., 2015; du Plooy et al., 2020), being employed (Elkeles & Seifert, 1996) and being less exposed to discrimination (Agudelo-Suárez et al., 2011; Williams & Mohammed, 2009) are associated with better health outcomes. Therefore, migrants from culturally similar countries are less likely to suffer the detrimental effects of acculturation, thus are more likely to have better morbidity outcomes. This is supported by previous evidence that migrants from ethnically and culturally similar countries have better health outcomes (e.g. self-rated health) compared with those with a different ethnic and cultural background in migrant-receiving nations such as Sweden (Wohland et al., 2015), Belgium, the Netherlands and England and Wales (Reus-Pons et al., 2017). In the present study, British migrants had the closest cultural similarities (in terms of customs, language, beliefs and diet) to the Australian-born population, while migrants from the other three European countries (Germany, Italy and Greece) also shared cultural similarities, albeit to a smaller extent. This advantage makes it easier for European migrants to migrate to Australia and adapt to the sociocultural environment compared with migrants from other cultural backgrounds. Additionally, the Australian Government encouraged the immigration of Europeans during specific periods (particularly before World War II), subsidising migration and settlement costs and granting free land to British and some European (particularly German) people to encourage them to migrate to Australia over destinations such as the US and Canada (Price, 1987). Under the slogan ‘Populate or perish’, the Australian Government also provided financial assistance (e.g. the Assisted Passage Migration Scheme) to a large number of refugees and displaced persons from Europe after World War II (National Museum Australia, 2022). While this meant that European migrants faced less financial pressure in their post-migration lives, it weakened the selectivity of European migrants. Consequently, the European migrants in this study (i.e. those from Britain, Germany, Italy and Greece) exhibited a less prominent healthy migrant effect in terms of LE but a greater effect in terms of HLE and the HLE/LE ratio compared with non-European migrants (i.e. those from China, India and Lebanon).

**Fig. 4:**
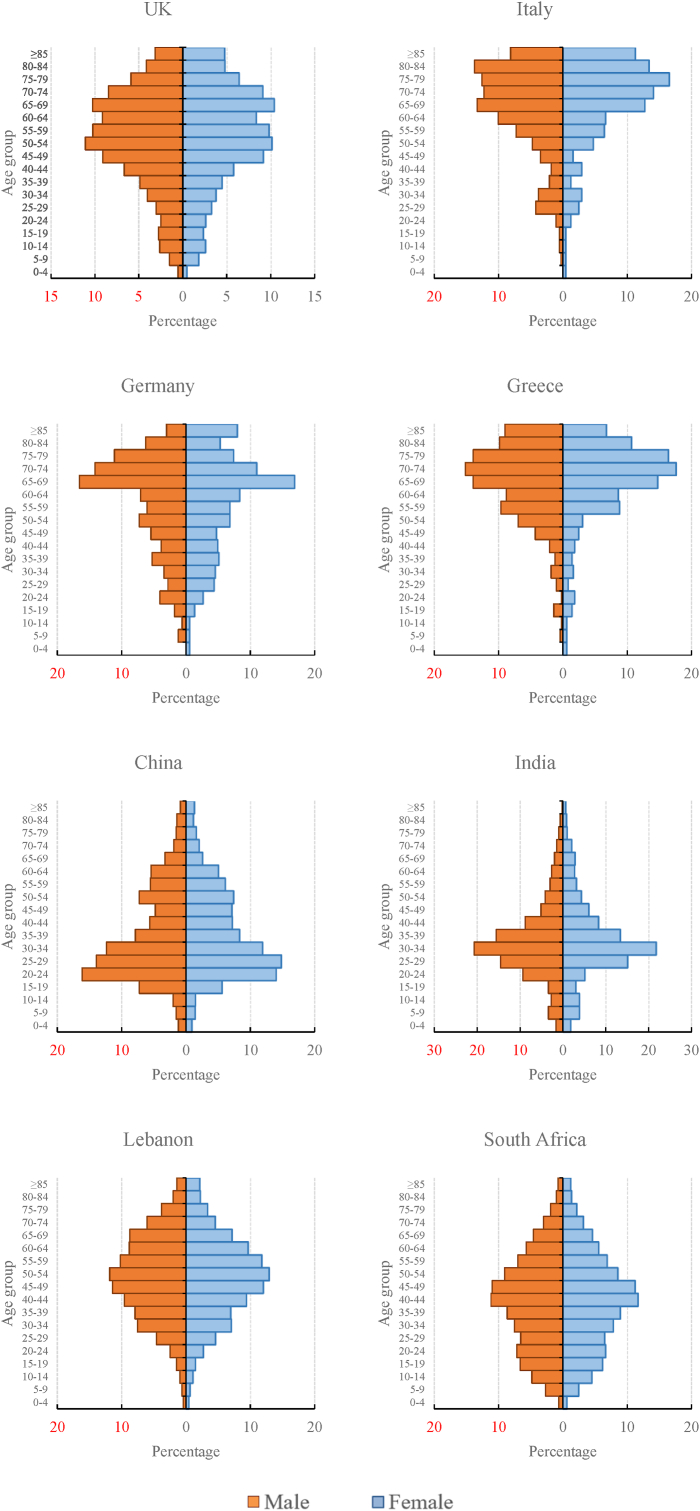
Age structures of selected major migrant groups in Australia, 2016

Second, the relationship between nativity and migrant health may also depend on duration of residence because health patterns in established migrant communities are likely to be similar to those of the native population (Biddle et al., 2007; McDonald & Kennedy, 2004). The main migration waves of the eight migrant groups in this study occurred during different historical periods. British, German, Italian and Greek migrant communities are well established in Australia, having been settled for more than five decades (see Table 1). In contrast, Chinese, Indian and South African migrants, who have a significantly younger age demographic (see Fig. 4), arrived much later, mostly in the 1980s after the White Australia policy had been officially abolished. With a longer duration of residence, migrants are more likely to integrate into mainstream society and adopt similar dietary habits and social behaviours, thus manifesting similar health outcomes to those of the host population. Major health indicators of longer-term migrants, such as rates of mortality (Ng, 2011) and prevalence of chronic disease (Biddle et al., 2007; McDonald & Kennedy, 2004), are similar to those of Australian-born individuals but different from those of recently arrived migrants. Therefore, in this study, established migrant communities, particularly those from the UK and Germany, exhibited similar LE and HLE outcomes to those of Australian-born individuals, while less-established migrant communities (i.e. Chinese, Indian and South African migrants) had substantially different LE and HLE outcomes. Lebanese migrants exhibited a distinct pattern from that of well-established migrant communities given their relatively recent wave of migration (with the majority arriving in Australia after the 1960s) and experience of being displaced by war and violence.

Third, the migration experiences of different migrant groups may be an important cause of varying health outcomes according to place of birth. This is particularly relevant to the outstanding health outcomes of South African migrants (who had the second highest LE and the highest HLE and HLE/LE ratio among the eight migrant groups) and the poor health outcomes of Lebanese migrants (the only group to experience a declining HLE and HLE/LE ratio from 2006 to 2016). Specifically, the majority of South African migrants in Australia are white, English speaking and of a European background (Wasserman, 2018), meaning that prior to migrating to Australia, South African migrants or their ancestors had migrated at least once (i.e. from Europe to South Africa). Some had even migrated multiple times, typically from Europe to northern African countries, then to South Africa following the wave of independence in many African countries (Mattes & Richmond, 2000). Given that the healthy migrant effect can be seen in both migrants and their descendants (Cunningham et al., 2008; Huijts & Kraaykamp, 2012), these multiple migrations may have enhanced South African migrant selectivity, contributing to better health outcomes. In addition, the existence of apartheid from 1948 to 1994 in South Africa, which created ethnic enclaves for white residents, may also have contributed to the desirable health outcomes of South African migrants because of the positive effect of ethnic enclaves in assisting migrants to seek social support and obtain employment (Edin et al., 2003; Guo et al., 2022). In contrast, the migration experiences of Lebanese migrants are largely associated with war and conflict. Almost 60% of Lebanese migrants arrived in Australia before 1990 (Australian Department of Home Affairs, 2020) (see Table 1), with most arriving as refugees fleeing the Arab–Israeli conflict in 1967 and the Lebanese Civil War in 1975 (Collins, 2005). These circumstances may have had adverse effects on Lebanese migrants' health outcomes. Studies have found that exposure to violence and war is a major threat to migrants' mental and physical health (Jasso et al., 2005), which may have contributed to the poorer health status of Lebanese migrants observed in this study. In addition, the overall downward trend in Lebanese migrants’ LE and HLE from 2006 to 2016 may be the result of a greater proportion of Lebanese migrants arriving after 1967. This may also explain the spike in LE for Lebanese migrants in the oldest age group, which corresponds with the low proportion of these migrants arriving after 1967 at this age group.

Fourth, the varying socioeconomic profiles of different migrant groups in Australia may help explain health disparities according to birthplace. Specifically, South African, British and German migrants in Australia have greater socioeconomic advantages compared with other migrant groups researched in this study (Wasserman, 2018). For example, 73.1% of South African migrants, 63.6% of British migrants and 69.2% of Germany migrants have a high school qualification or above compared with 60.1% of the total Australian population, 37.2% of Lebanese migrants, 36% of Italian migrants and 25.8% of Greek migrants (Australian Department of Home Affairs, 2020). Similarly, the weekly salary of South African, British and German migrants is AU$984, AU$709 and AU$534, respectively, significantly exceeding that of many other migrant groups (e.g. AU$374 for Chinese migrants, AU$408 for Lebanese migrants, AU$435 for Italian migrants and AU$392 Greek migrants). Moreover, while 62.4% of South African migrants, 56.0% of British migrants and 57.2% of German migrants are employed in either a skilled managerial, professional or trade occupation, the corresponding rate is notably lower in other migrant groups (e.g. 45.5% for Greek migrants) (Australian Department of Home Affairs, 2020). In contrast, the unemployment rates among South African, British and German migrants are considerably lower than that of other major migrant groups (Australian Department of Home Affairs, 2020) (see Table 1). High socioeconomic status is associated with desirable health outcomes, while low socioeconomic status is associated with poorer health outcomes (Sicular et al., 2007). Therefore, socioeconomic differences among migrant groups may contribute to migrants’ LE and HLE disparities according to birthplace.

Fifth, the changing socioeconomic profiles of the countries of origin of the eight selected migrants during the study period may also have led to the health disparities in different migrant groups. This is particularly significant for Chinese and Indian migrants, whose LE and HLE improved at a faster pace from 2006 to 2016 compared with that of the overall foreign-born population. Specifically, China and India experienced a significant increase in economic development from 2006 to 2016, with their total gross domestic product (GDP) increasing dramatically from US$2.8 to US$11.2 trillion and US$0.9 to US$2.3 trillion, respectively, and GDP per capita increasing from US$2099 to US$8094 and US$802 to US$1,714, respectively (World Bank, 2023). In addition, the healthcare expenditure per capita of these two countries also increased from US$81.2 to US$395.4 and US$29.6 to US$60.6, respectively (World Bank, 2023). The rapid economic development and the significantly higher healthcare expenditure may contribute to improved health outcomes for the Chinese and Indian populations, thus the improved overall LE and HLE of migrants from these two countries.

Noteworthily, the age variations of the nativity difference in the three health indicators, particularly LE and HLE, echo the theory of age-as-leveler. The narrowed health differences by nativity with age, observed in this study, suggest that as age increases, the effect of sociocultural differences on the health disparities decreases (Dupre, 2007; Ferraro & Farmer, 1996). The decreased health differences by nativity might be explained by the selective mortality, a process whereby socioeconomically disadvantaged individuals die at younger ages than their more advantaged peers (Beckett, 2000). They might also be explained by the social welfare programs designed to improve the wellbeing and health outcomes of old adults. In Australia, social welfare programs, including Age Pension, Aged Care Subsidy and Pensioner Concession Card, are in place to support the basic living standards of older Australians and tend to provide more subsidy for socioeconomically disadvantaged older adults. These programs ensure all older Australians, regardless of their sociocultural backgrounds, can access basic health and aged care, and hence, help reduce the health disparities by nativity among older Australians.

### 5.4 Policy implications

The findings of this study have several important policy implications for Australia and elsewhere. First, given the growing number of migrants and the fact that most migrants have increased rates of morbidity in middle and old age, policymakers and care providers should be aware of the increasing demands for health care from migrant communities. Thus, in addition to conventional efforts to reduce mortality rates, a greater focus on improving migrants' quality of life is needed. Second, given the considerable variations in the association between nativity and migrant health outcomes, identifying vulnerable migrants will be important to improve the allocation of healthcare resources. In particular, migrants from countries that are culturally different from Australia merit increased attention. Supporting migrants from different cultural backgrounds to better integrate into society, including eliminating discrimination, encouraging multiculturalism and narrowing socioeconomic inequalities within the migration population, will be helpful. Third, refugees' unfavourable health outcomes resulting from exposure to war and conflict can last for decades. Special attention should be given to refugees’ health status given that the number of refugees, both domestically and internationally, has increased in recent years.

### 5.5 Limitations

This study has some limitations. First, by using the Sullivan method, it is assumed that mortality and morbidity rates for specific years represent that which is actually experienced by real people over the course of their lives. LE and HLE computed in this study were based on data from overlapping generations in a specific period rather than on data from real cohorts. Longitudinal studies are recommended to explore changes in migrant health and wellbeing over time if such data become available. Second, the effect of gender on migrant health outcomes in relation to birthplace was not investigated. This was to ensure the robustness of the results given that the number of deaths at 45–54 years is relatively small if divided by gender. Future researchers could combine mortality data from several sequential years or use a higher starting age (e.g. 65 years) to solve this problem. Third, this study focused on physical rather than mental health. Quality of life should be investigated comprehensively by considering both physical and mental health to better capture the wellbeing of migrants. Fourth, this study adopted a combined measure of health based on three dimensions—self-care, mobility and communication—distinguishing it from previous studies that have overlooked communication ability when measuring health status. The CPH measures health according to individuals' care needs, which are crucially affected by a care recipient's communication ability; thus, the inclusion of communication ability helps to better capture changing care needs with age. However, it is noteworthy that while ABS claims that communication difficulties due to English proficiency are not considered as disability in CPH, language barriers, such as lack of English proficiency, do affect individuals' ability to access care resources to meet their care need, particularly for those with dementia that might compromise ability of using English as a second language during ageing process (Ellajosyula et al., 2020; Potkins et al., 2003; Tsoh et al., 2016). Additionally, because the CPH does not provide further measures of these three dimensions separately, a more granular analysis of the three core activities was not conducted. Fifth, the eight migrant groups examined in this study were included in the sample regardless of year of arrival. It would be interesting to explore the differences in LE and HLE by country of birth and duration of residence, which could better capture migrants' health disparities by country of birth at different stages of the acculturation process. However, given the lack of data, particularly with respect to the age-specific mortality rates of migrants by year of arrival (overall and by country of birth), this is not currently possible.

Despite these limitations, this study provides comprehensive insights into mortality and morbidity among eight major Australia migrant groups and the Australian-born population, providing a new understanding of the variations in the healthy migrant effect among different migrant groups. The results from this study enrich the understanding of migrant HLE and provide new insights into migrant health disparities, which may contribute to better policies on healthcare provision and enhanced quality of life outcomes for migrants in Australia and elsewhere.

## Funding

This work was supported by 10.13039/501100000923Australian Research Council Discovery Project- Demographic and Social Dimensions of Migrant Ageing and Wellbeing in Australia (grant number DP190102778). The funding organisation did not have any influence on the study design, data collection, analysis and interpretation as well as the preparation, review, or approval of the manuscript for publication.

## Ethical statement

The study was based on data from census in Australia so no separate approval was required for this study.

## Author contributions

G.H. and F. G. conceptualised and designed the study. G.H. acquired and analysed the data with input from F.G., Z.C., and M.T. in methodology. G.H. wrote the first draft of the article under the supervision of F.G. and Z.C. K.Z, L.L and M. F critically revised the draft. All authors reviewed and approved the final version of the article. F.G. and L.T. coordinated research activities and led the team.

We state that this paper has not been published or submitted for publication elsewhere, and we acknowledge that all authors have contributed significantly and that all authors are in agreement with the content of the manuscript.

## Declaration of competing interest

The authors have no competing interests to declare that are relevant to the content of this article.

## Data Availability

The authors do not have permission to share data.
